# Dr. Henry Herbert Herbst

**Published:** 1911-11

**Authors:** 


Dr. Henry Herbert Herbst, University of Pennsylvania, 1884, ex-mayor of Allentown, a member of the A.M.A., American Academy of Medicine, a man who had held many positions of honor and who was a pioneer in the medical inspection of school children, died of nephritis, at the University Hospital, Philadelphia, September 20, 1911, aged 53.

